# ChatGPT in cardiovascular medicine: revolution, hype, or helper?

**DOI:** 10.3389/fpubh.2025.1622561

**Published:** 2025-09-10

**Authors:** Izabella Uchmanowicz, Maria Jędrzejczyk, Ercole Vellone, Sara Janczak, Karol Mirkowski, Bartosz M. Uchmanowicz, Michał Czapla

**Affiliations:** ^1^Department of Nursing, Faculty of Nursing and Midwifery, Wroclaw Medical University, Wroclaw, Poland; ^2^Centre for Cardiovascular Health, Edinburgh Napier University, Sighthill Campus, Edinburgh, United Kingdom; ^3^Department of Biomedicine and Prevention, University of Rome Tor Vergata, Rome, Italy; ^4^Student Scientific Association, Wroclaw Medical University, Wroclaw, Poland; ^5^Faculty of Electronics, Photonics and Microsystems, Wroclaw University of Technology, Wroclaw, Poland; ^6^Group of Research in Care (GRUPAC), Faculty of Health Sciences, University of La Rioja, Logroño, Spain; ^7^Division of Scientific Research and Innovation in Emergency Medical Service, Department of Emergency Medical Service, Faculty of Nursing and Midwifery, Wroclaw Medical University, Wroclaw, Poland

**Keywords:** artificial intelligence, cardiovacular diseases, evidence-based practice, ChatGPT, clinical decision-making

## Abstract

The integration of artificial intelligence (AI) into healthcare has opened new frontiers in clinical research and practice, particularly in data-rich disciplines like cardiovascular medicine. Among recent advancements, ChatGPT—a large language model developed by OpenAI—has garnered increasing attention for its potential to streamline workflows, support literature synthesis, and facilitate data interpretation. This review examines the multifaceted applications of ChatGPT in cardiovascular medicine, including its use in hypothesis generation, research design, evidence-based decision-making, and patient communication. ChatGPT offers the ability to process and summarize large volumes of medical literature and patient data, potentially enhancing the efficiency and accessibility of research activities. It can also assist in creating patient-friendly educational materials and support patient-centered care through more personalized communication. However, the use of generative AI models in clinical research raises critical concerns related to the accuracy of generated content, ethical implications, and the absence of contextual clinical judgment. Limitations such as hallucinations, data privacy issues, and the risk of overreliance on non-human decision-making must be addressed through rigorous oversight, validation, and clear guidelines for responsible use. While not a substitute for human expertise, ChatGPT can act as a valuable complementary tool that enhances research productivity and innovation in cardiovascular medicine. By supporting clinicians and researchers in navigating complex datasets and rapidly evolving evidence, ChatGPT holds promise as a facilitator of more efficient, inclusive, and responsive cardiovascular care—provided its integration is approached with caution and critical appraisal.

## 1 Introduction

Cardiovascular medicine is a rapidly evolving field that relies heavily on evidence-based research to address the complex, multifactorial needs of patients. The ever-increasing volume of scientific publications, clinical guidelines, and patient data presents both opportunities and challenges for researchers and clinicians.

To keep pace, healthcare professionals are increasingly exploring—and initiating discussions in the recent literature about—digital tools that can streamline workflows, support data interpretation and enhance the design of patient-centered interventions (1–5). Among these emerging technologies, artificial intelligence (AI)—and in particular large language models (LLMs) such as ChatGPT (Generative Pretrained Transformer)—has garnered considerable attention (6). ChatGPT, developed by OpenAI, is a generative AI model trained on extensive textual datasets and designed to produce human-like responses across a range of prompts. It is worth noting that the term “ChatGPT” refers to an evolving interface that has been powered by various large language models, including GPT-3, GPT-3.5, GPT-4, and the most recent GPT-4o (released in 2024). Each successive model has shown improvements in reasoning, contextual understanding, and domain-specific performance, particularly in medicine. However, as the cited literature spans different periods of model availability, not all referenced studies are based on the same version. Therefore, generalizations about ChatGPT’s capabilities should be interpreted in the context of ongoing model evolution. Across its iterations, ChatGPT has demonstrated functionalities including language comprehension, synthesis of textual information, and generative capabilities that may support clinical documentation, evidence retrieval, patient communication and hypothesis generation (7). Within cardiovascular medicine—where decision making often requires integration of diverse data streams, interpretation of evolving evidence and communication with multidisciplinary teams—technologies such as ChatGPT offer innovative solutions to manage and analyze data efficiently (8).

As noted by Drazen et al., AI chatbots like GPT-4 are sensitive to the wording of prompts, giving rise to the concept of “prompt engineering” (9). While AI systems typically provide correct responses to prompts with definitive answers, their outputs can become problematic when faced with ambiguous questions, leading to inaccuracies known as “hallucinations.” These errors can be particularly dangerous in medical scenarios because the inaccuracies can be subtle yet convincingly stated, potentially misleading clinicians and impacting patient care negatively. Hence, it is crucial for healthcare professionals to verify AI-generated information carefully and not rely solely on chatbot outputs (9). AI’s role in healthcare has been transformative, enabling large-scale data processing and pattern recognition, which support personalized care and informed decision-making (10–12). This review explores ChatGPT’s applications in cardiovascular care, highlighting its advantages in supporting research while also addressing the ethical and practical challenges associated with AI use in clinical contexts.

## 2 Applications of ChatGPT in cardiovascular research

### 2.1 Literature review and evidence synthesis

Conducting literature reviews is foundational in cardiovascular medicine, supporting clinical decision making and the development of evidence based protocols. However, the process is time-consuming and resource-intensive, particularly as the volume of published medical research continues to grow exponentially. In recent years, AI has been used effectively to automate data synthesis and literature review, offering researchers timely insights into evolving clinical practices (13).

ChatGPT enhances this process by summarizing findings from extensive cardiovascular datasets, clinical guidelines, and evidence-based practices, enabling researchers to quickly access critical information. This automation supports consistency in evidence-informed practice by aligning research with the latest standards in cardiovascular care (14–16).

As noted in recent studies, large language models (LLMs) like ChatGPT are increasingly acceptable tools in academic and clinical medicine due to their ability to handle large volumes of information while providing reliable summaries and identifying research gaps (Kim JK, Chua M, Rickard M, Lorenzo A). ChatGPT and LLM chatbots: The current state of acceptability and a proposal for guidelines on utilization in academic medicine (17–19). This aligns with precision medicine goals by encouraging targeted research based on unmet needs in specific demographics, such as understudied cardiovascular conditions or populations affected by health disparities (20).

### 2.2 Data analysis and interpretation

Data analysis and interpretation are central to cardiovascular medicine, helping researchers and clinicians to evaluate patient outcomes, identify treatment response patterns and assess the effectiveness of interventions. ChatGPT aids this process by conducting preliminary analyses of patient data from electronic health records (EHRs) to identify trends that may affect cardiovascular health, recovery, and readmission rates (10, 21). AI has demonstrated success in analyzing both structured patient data (e.g., lab values, vital signs) and unstructured patient data (e.g., clinician notes), enhancing the predictive accuracy of clinical outcomes and potentially improving patient care (2, 22).

ChatGPT’s ability to rapidly process large datasets offers an opportunity to assist in reviewing patient histories and synthesizing multi-visit records—helping with the tasks that are often time consuming.

Lüscher et al. discuss AI’s transformative role in analyzing patient data to enhance diagnostic and prognostic accuracy. This approach may allow clinicians to better understand patient experiences, feedback, and adherence, fostering a deeper level of insight into patient-centered research outcomes (15, 23). Recent studies have demonstrated tangible applications of artificial intelligence in cardiovascular practice. For instance, Amadio et al. developed an AI-enhanced electrocardiogram (ECG) model that accurately predicted mortality risk among patients with cardiac amyloidosis by using a diagnostic A2E score. This score independently stratified prognosis in both AL and ATTR amyloidosis, offering actionable insight for clinical decision-making (24). Similarly, Shao et al. applied machine learning to electronic health records of over 600,000 U. S. veterans to phenotype heart failure patients. Their model outperformed traditional clustering methods, identifying novel clinical subtypes that may inform personalized treatment strategies (25). In another recent study, Weller et al. used AI models trained on routine e-health records to predict 90-day mortality in older adults patients hospitalized with acute heart failure (26). Their model achieved high predictive accuracy and highlighted the potential of AI to support early risk stratification in vulnerable cardiovascular populations. Collectively, these studies illustrate how AI—when grounded in robust clinical data—can contribute to more personalized and proactive cardiovascular care.

### 2.3 Hypothesis generation and research design

Hypothesis generation is a critical phase of research, shaping study objectives, influencing methodology and guiding data interpretation. In cardiovascular medicine—where the interplay of genetic, behavioral and clinical variables is often complex—formulating meaningful, testable hypotheses requires a nuanced understanding of multifactorial datasets. The role of AI in this process has been increasingly recognized, with models capable of identifying latent patterns and suggesting novel research directions based on large scale data analyses (13).

ChatGPT may assist researchers during the early stages of inquiry by helping to formulate research questions derived from existing literature and previously identified gaps in evidence. Its ability to summarize large volumes of scientific text, identify thematic patterns and propose logical associations can support the iterative refinement of research aims, particularly when used alongside human judgment. Alowais et al. emphasize the broader potential of AI in clinical research planning, noting its contributions to hypothesis generation and protocol drafting across medical disciplines (12). According to Moons and Van Bulck, ChatGPT’s utility in formulating research questions and suggesting appropriate methodologies provides researchers with a starting point for addressing complex patient-centered inquiries (3). Additionally, Kim et al. propose that establishing usage guidelines for ChatGPT in research ensures its effective integration, particularly in high-impact fields such as cardiology where both rigor and relevance to patient care are essential (19).

### 2.4 Patient education and support

Patient education is fundamental in cardiovascular care, as informed patients are more likely to engage in their care and adhere to treatment plans. AI technologies—including large language models like ChatGPT—has been effective in patient education, with applications that provide patients with tailored information on disease management and health behaviors (20, 23). ChatGPT supports this role by creating simplified educational materials that explain complex medical concepts, making information more accessible to patients. Its multilingual and conversational interface may also enhance accessibility for diverse patient populations.

**Figure 1:**
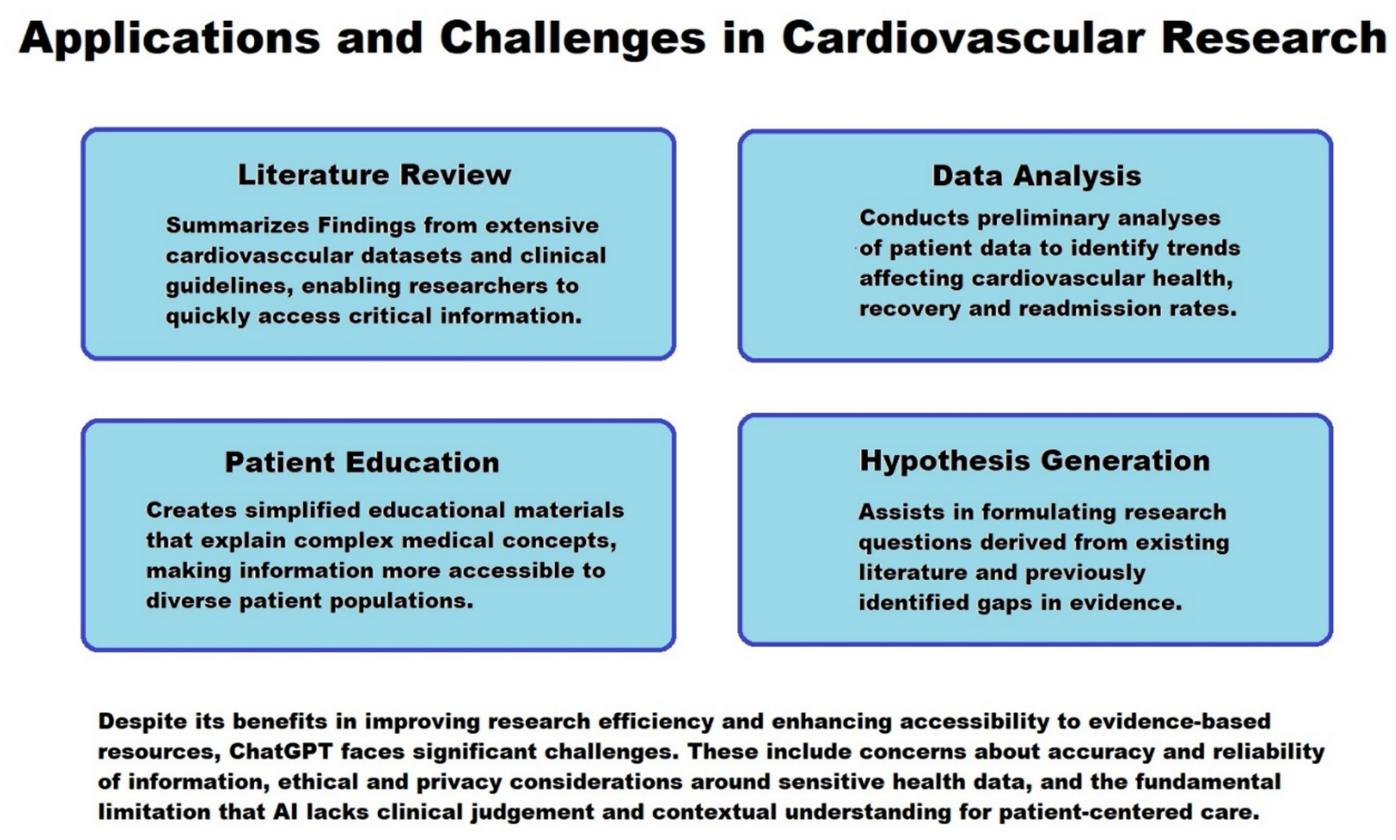
Application and challenges in cardiovascular research.

Fink et al. emphasizes the patient-centered potential of ChatGPT in creating resources that are culturally sensitive, accessible, and tailored to individual patient needs (18). In cardiovascular medicine, where outcomes are often influenced by socioeconomic and demographic factors, such personalization may contribute to reducing disparities in health education. By assisting healthcare professionals in delivering consistent and comprehensible information, ChatGPT has the potential to enhance patient engagement and satisfaction, provided that the content is reviewed and approved by clinicians (16, 23). Figure 1 summarizes application and challenges in cardiovascular research.

## 3 Advantages of using ChatGPT in cardiovascular research

### 3.1 Improved research efficiency

One of ChatGPT’s main benefits is its ability to streamline time-intensive tasks such as literature synthesis, preliminary data processing and survey analysis. In cardiovascular research, this increased efficiency enables researchers to concentrate on data collection and patient care. By expediting repetitive tasks, ChatGPT supports the delivery of timely, evidence-based findings that inform clinical practice and improve patient outcomes (3, 11).

### 3.2 Enhanced accessibility to evidence-based resources

Evidence-based practice is essential in cardiovascular care, but keeping up with new research can be challenging due to the overwhelming volume of publications. ChatGPT’s summarization capabilities make it easier to access and apply current best practices, supporting consistent, high-quality care. In clinical settings where time and resources are limited, this quick access to synthesized information can support timely and informed decision-making (27). Moons and Van Bulck emphasize that ChatGPT’s role in delivering summaries and evidence synthesis aligns well with the need for rapid access to updated clinical guidelines, helping to ensure high standards in care (3). Furthermore, the tool’s interactive nature enables users to ask follow-up questions, clarify complex topics, or focus on specific subtopics within cardiovascular medicine, which enhances its educational value for both clinicians and trainees.

### 3.3 Cost reduction

ChatGPT reduces costs in cardiovascular research by automating routine tasks, reducing the need for extensive manpower in areas such as literature review and preliminary data processing—which typically require substantial personnel time. This cost-saving potential allows researchers to focus resources on more complex aspects of patient care and research design, thus making advanced studies more financially feasible (16, 18). In addition, minimizing reliance on manual data extraction or repetitive documentation tasks can improve operational efficiency in both academic and clinical environments. For institutions with limited funding, ChatGPT offers a low-cost solution to support research continuity and maintain high-quality outputs without compromising scientific rigor.

### 3.4 Enhanced patient-centered research

**Figure 2:**
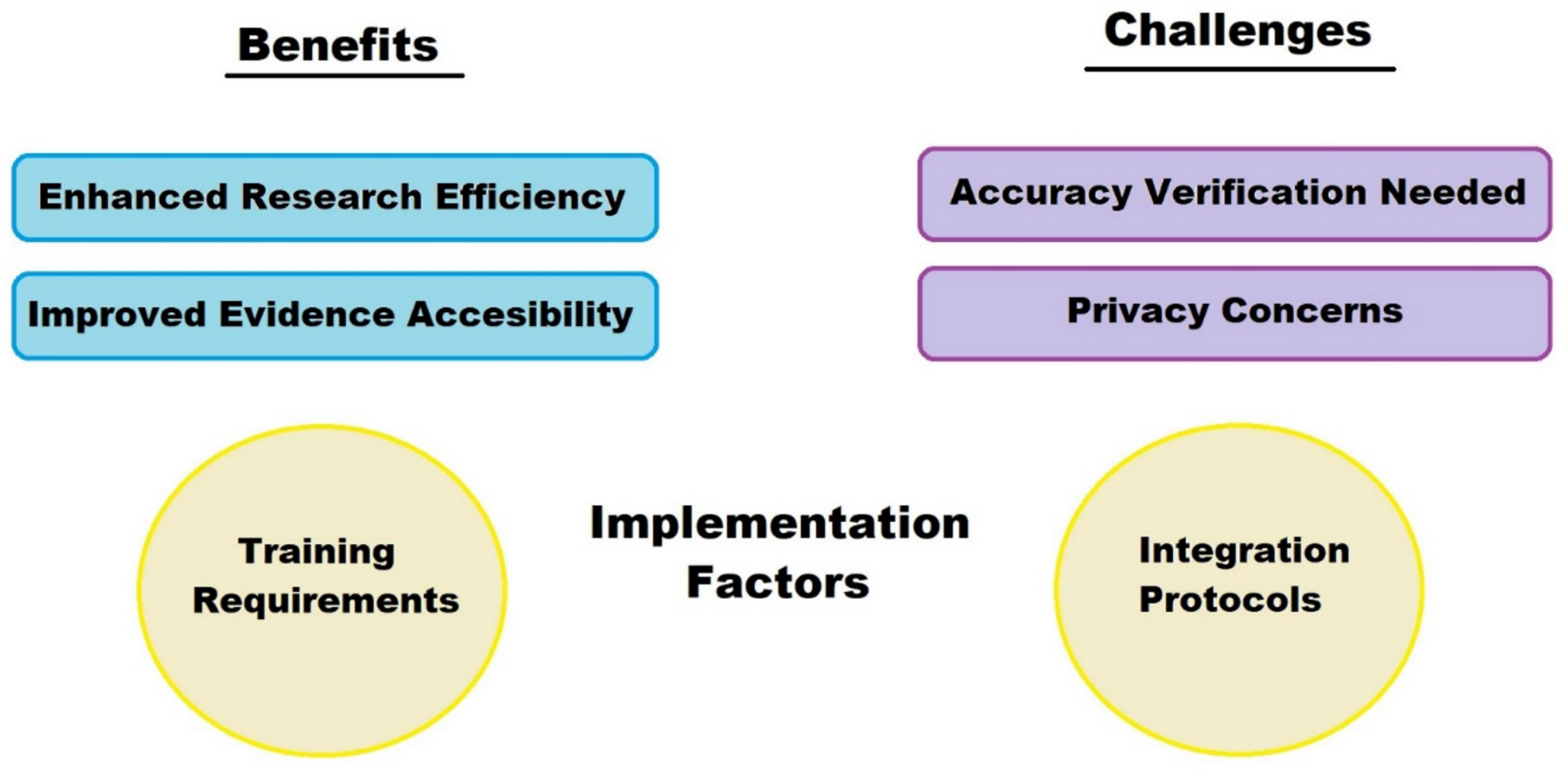
Benefits vs. challenges metrics. The figure illustrates the dual aspects of ChatGPT’s role: enhancing research efficiency and evidence accessibility (top left), while highlighting the need for accuracy verification and data privacy (top right). Practical implementation also requires attention to training and integration protocols (bottom).

ChatGPT’s role in creating patient education materials and analyzing patient-reported outcomes aligns with the shift toward patient-centered research. By supporting research that respects patient experiences and needs, ChatGPT contributes to improved patient engagement and adherence, aligning with AI’s broader potential to individualize healthcare and enhance patient satisfaction (13). Moreover, its ability to process free-text data—such as open-ended survey responses, feedback forms, or online patient forums—enables researchers to capture nuanced perspectives that may be overlooked in structured datasets. This qualitative insight can inform the development of more tailored interventions, especially in cardiovascular care where psychosocial factors and lifestyle considerations play a significant role. In addition, ChatGPT can assist in co-creating communication materials with patients, ensuring clarity, accessibility, and cultural sensitivity in health education (28, 29). To complement the discussion on the benefits and limitations of ChatGPT in cardiovascular research, Figure 2 summarizes key advantages, associated challenges, and practical implementation considerations.

## 4 Challenges and limitations

### 4.1 Accuracy and reliability of information

Despite its utility, AI models, including ChatGPT, occasionally generate inaccurate, misleading or irrelevant responses—a phenomenon known as “hallucination,” which can undermine research quality in fields requiring high precision. It is particularly concerning because the errors produced by the chatbot can be nuanced and difficult to detect right away, yet are often delivered with such fluency and confidence that users may mistakenly perceive them as accurate and trustworthy. Cardiovascular research relies on accurate data interpretation, and errors in AI outputs could impact patient care. AI’s limitations in this regard underscore the need for careful oversight, as previously noted in studies emphasizing the importance of validation in clinical AI applications (3, 7, 10, 15, 20). Without a built-in mechanism for real-time source attribution or cross-verification, ChatGPT should not be relied upon as a standalone reference tool.

### 4.2 Ethical and privacy concerns

AI’s application in healthcare raises ethical concerns, especially regarding patient data privacy. The protection of sensitive health data is critical in cardiovascular care, and ChatGPT’s data processing protocols must align with HIPAA, GDPR, and other privacy regulations to avoid breaches. Ethical use of AI in healthcare demands transparency and robust data security measures that uphold patient confidentiality (2, 19, 30). Beyond general compliance, additional challenges emerge when ChatGPT or similar AI models are integrated with electronic health records (EHRs) or cloud-based infrastructures. These systems must ensure data anonymization and implement access controls, encryption, and activity logging (audit trails) to prevent unauthorized access and trace potential data misuse. Furthermore, healthcare professionals must be adequately trained to avoid inadvertently sharing identifiable patient information when interacting with AI systems. Special consideration should also be given to the geographic location of servers used for data processing, as cross-border data transfers may conflict with local data sovereignty laws.

### 4.3 Lack of clinical judgment and contextual understanding

Although ChatGPT is effective at processing large amounts of data, it lacks the clinical reasoning, situational awareness and empathy that characterize patient-centered care. Cardiovascular medicine requires an understanding of patient-specific variables that ChatGPT may not fully capture, making human oversight crucial and irreplaceable (31). For example, subtle clinical cues such as patient affect, tone of voice, or physical examination findings remain outside the model’s scope, yet these often guide nuanced decision-making. AI should therefore be viewed as a supplementary tool, with experienced professionals ensuring its outputs align with patient-centered principles (3, 11).

### 4.4 Practical and public health implications

While much of the current discourse on ChatGPT in cardiovascular medicine focuses on individual clinical or academic use, its broader public health potential deserves equal attention. LLMs could serve as scalable tools in resource-limited settings by supporting health education, triage, and remote consultations—particularly for patients with limited access to cardiovascular specialists. In multilingual regions, ChatGPT’s translation and summarization capabilities may help bridge language barriers, enabling culturally sensitive, community-tailored communication that promotes prevention and treatment adherence.

Moreover, when embedded within broader digital ecosystems, LLMs could complement population-level cardiovascular risk surveillance and intervention strategies. For example, Singapore’s recent integration of AI-powered platforms like CardioSight and CHAMP into national EMRs demonstrates how real-time risk stratification, geospatial data visualization, and automated patient messaging can be used to close prevention gaps and reduce disease burden across large populations. These precedents suggest that the future role of generative AI may extend well beyond academic or research contexts, supporting national strategies for cardiovascular health equity, targeted screening, and early intervention in high-risk communities (32). These developments carry particular relevance for public health priorities in low-resource and underserved settings. By providing cost-effective, on-demand support for patient education, triage, and clinical decision-making, tools like ChatGPT may alleviate workforce shortages and enhance access to cardiovascular care. Furthermore, their ability to deliver information in multiple languages and adapt content to varying literacy levels directly supports digital health equity—especially among marginalized or linguistically isolated populations. This may be particularly relevant in the Asia–Pacific region, where cardiovascular diseases remain the leading cause of death, and health systems face challenges related to workforce shortages, geographical disparities, and digital access gaps (33).

## 5 Conclusion

ChatGPT offers substantial potential for cardiovascular medicine, enhancing research efficiency, supporting evidence-based practice, and contributing to more personalized patient-centered care. Its ability to streamline literature synthesis, assist with hypothesis generation, and facilitate patient education can be particularly valuable in high-demand clinical and academic environments. However, its application must be managed responsibly to address limitations in accuracy, ethics, and clinical judgment necessitate its use as a supplementary tool rather than a standalone solution.

When implemented responsibly and supported by human expertise, ChatGPT can complement the work of cardiovascular researchers, educators and clinicians. It offers a framework for accelerating innovation, improving communication and broadening access to high-quality medical knowledge. By complementing human expertise rather than attempting to replace it, ChatGPT can help advance a more informed, accessible and responsive future for cardiovascular care.
